# The Influence of Molecular Factors on the Effectiveness of New Therapies in Endometrial Cancer—Latest Evidence and Clinical Trials

**DOI:** 10.3390/cancers18030356

**Published:** 2026-01-23

**Authors:** Wiktoria Mytych, Edyta Barnaś, Dorota Bartusik-Aebisher, David Aebisher

**Affiliations:** 1 English Division Science Club, Faculty of Medicine, Collegium Medicum, The Rzeszów University, 35-310 Rzeszów, Poland; wiktoriamytych@gmail.com; 2 Institute of Health Sciences, Faculty of Medicine, Collegium Medicum, The Rzeszów University, 35-959 Rzeszów, Poland; ebarnas@ur.edu.pl; 3 Department of Biochemistry and General Chemistry, Faculty of Medicine, Collegium Medicum, The Rzeszów University, 35-310 Rzeszów, Poland; dbartusikaebisher@ur.edu.pl; 4 Department of Photomedicine and Physical Chemistry, Faculty of Medicine, Collegium Medicum, The Rzeszów University, 35-310 Rzeszów, Poland

**Keywords:** endometrial cancer, immunotherapy, genetic, targeted therapy, molecular, chemotherapy, hormone therapy

## Abstract

Endometrial cancer (EC) is the most common gynecological cancer in developed countries, with approximately 417,000 new cases reported worldwide in 2020. Its incidence has been rising for the past 30 years, primarily due to population aging, obesity, and type 2 diabetes; obesity accounts for almost half of cases due to excessive estrogen production. The classic division into types I and II was replaced in 2013 by the molecular TCGA classification, which distinguishes four subtypes: POLE-ultramutated (best prognosis), MSI-hypermutated, copy-number low, and copy-number high (worst prognosis). This classification (refined in ProMisE and TransPORTEC) enables precise treatment: immunotherapy (pembrolizumab, dostarlimab) works excellently in dMMR/MSI-H tumors, PI3K/AKT/mTOR inhibitors and trastuzumab deruxtecan in selected molecular subtypes, and hormone therapy in ER-positive tumors. ctDNA monitoring supports therapeutic decisions. Integrating the molecular profile with FIGO allows for truly personalized treatment, although MMRp/MSS tumors remain a challenge. The future lies in multi-omics, new biomarkers, and combination therapies.

## Simple Summary

Endometrial cancer, a common type affecting the womb lining, is on the rise due to factors like aging populations and obesity, making it urgent to improve treatment success. This review examines how specific molecular and genetic traits in tumors influence the effectiveness of emerging therapies, such as immune-boosting drugs, targeted treatments, and hormone options, with the goal of enabling more personalized care for patients. By highlighting these connections, the findings could guide scientists and doctors toward better-tailored strategies, potentially enhancing survival rates and inspiring new studies on innovative biomarkers and combined approaches to tackle this disease more effectively.

## 1. Introduction

**Figure 1:**
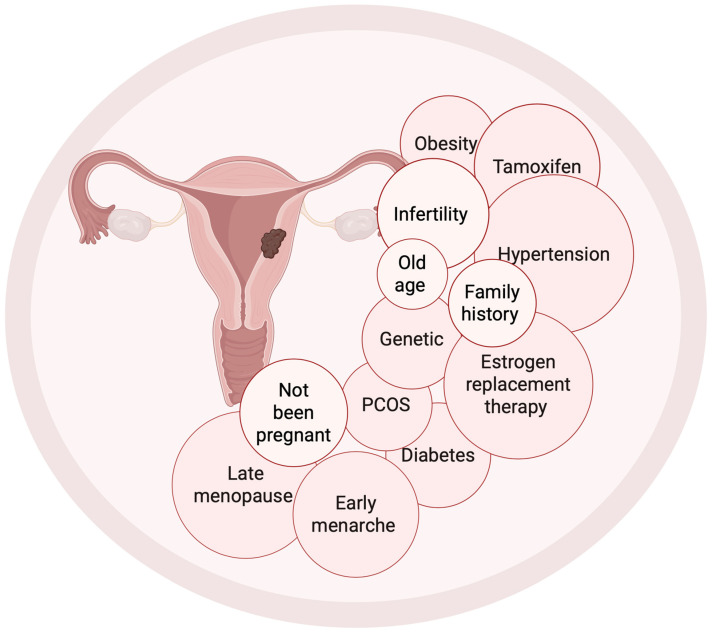
Presents a diagram of the uterus with labeled risk factors for endometrial cancer. Created with BioRender.com.

Endometrial cancer (EC) is the most common gynecological cancer in developed countries, and there has been a steady increase in the incidence of EC worldwide. According to GLOBOCAN data from 2020, approximately 417,000 new cases were reported worldwide, placing EC sixth among all malignant cancers in women [1]. In the United States, EC is the fourth leading cause of cancer deaths among women, with approximately 12,000 patients diagnosed annually [2]. Over the past 30 years, the incidence of EC has been on the rise, mainly due to an aging population (Figure 1), increasing obesity, type 2 diabetes, and hormonal disorders [3,4]. Obesity accounts for nearly half of EC cases, increasing the risk primarily through excessive estrogen production in adipose tissue [5].

Postmenopausal bleeding is the most common presenting symptom of endometrial cancer, enabling diagnosis at early stages (FIGO I–II) in most cases, with a 5-year overall survival rate of 80–90% [6]. In contrast, survival in advanced stages (III–IV) drops below 20–40%, depending on the series. Risk factors include advanced age, postmenopausal status, polycystic ovary syndrome (PCOS), unopposed estrogen exposure (e.g., estrogen-only hormone replacement therapy or obesity), diabetes, and hypertension [7,8]. Hereditary predisposition accounts for approximately 3–5% of cases, most notably Lynch syndrome caused by germline mutations in DNA mismatch-repair (MMR) genes [9,10].

### 1.1. Classification of Endometrial Cancer

Historically, endometrial cancer has been divided into two main histological types: type I (endometrioid, estrogen-dependent, and with a better prognosis) and type II (non-endometrioid, serous or clear cell, and more aggressive) [11]. Type I (80% of cases) is characterized by mutations in the *PTEN*, *KRAS*, and *CTNNB1* genes and microsatellite instability (MSI) [12]. Type II is most associated with *TP53* mutations, a high Ki-67 index, HER2 amplification, and a significantly worse survival prognosis [13]. The 2009 FIGO classification, updated in 2023, is based on clinical stage and risk of EC recurrence. It distinguishes between low (non-invasive), intermediate (<50% myometrial invasion), and high (50% or more invasion or no endometrioid features) stages [14]. However, this classification has limitations, low repeatability of histological assessment, and a lack of precise prediction of response to treatment. Studies from 2020–2025 show that in 20–30% of patients, the traditional classification does not reflect molecular heterogeneity, leading to excessive or insufficient treatment. A breakthrough came with the molecular classification developed by The Cancer Genome Atlas (TCGA) [15], which integrates genetic and histological data.

### 1.2. Molecular Evolution and Classification of TCGA

**Figure 2:**
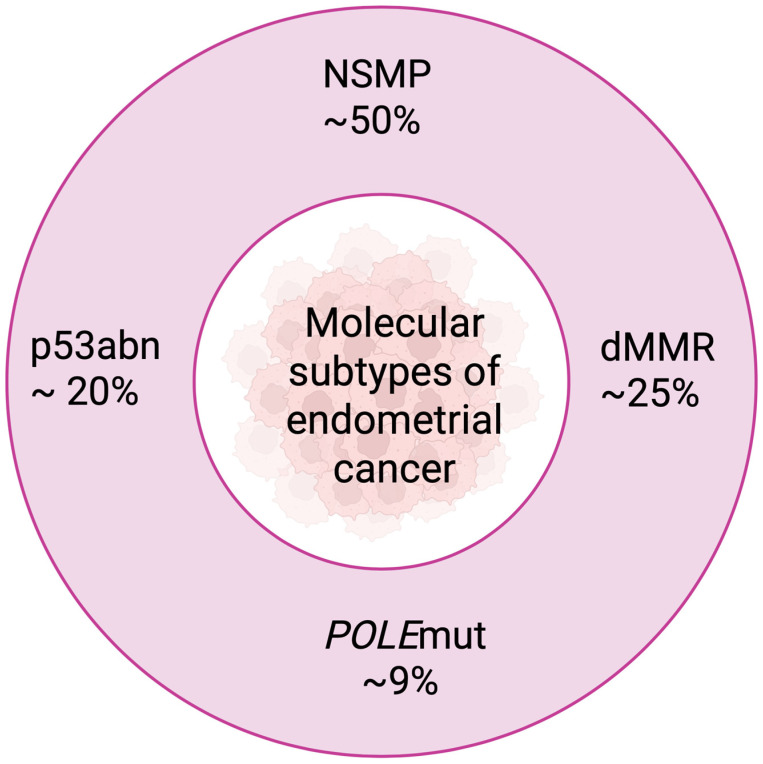
Molecular classification of endometrial cancer according to the TCGA classification. The most common subtype is NSMP (no specific molecular profile)—approx. 50%, followed by dMMR (mismatch repair deficient)—approx. 25%, p53abn (p53 abnormal)—approx. 20%, and POLEmut (POLE ultramutated)—approx. 9%. Created with BioRender.com.

In 2013, based on an analysis of 373 cases, the TCGA identified four main molecular subtypes of EC (Figure 2) [16]. These were the POLE ultra-mutated type (best prognosis), the MSI hypermutated type (intermediate prognosis), the CNL type (low number of somatic mutations, good prognosis), and the CNH type (high number of mutations and changes in copy numbers, aggressive, poor prognosis) [1,17]. Further work by ProMisE (Proactive Molecular Risk Classifier of Endometrial Cancer) and TransPORTEC simplified the classification into four diagnostic categories that can be determined in routine practice on FFPE material. These include POLEmut (mutations in the *POLE* gene), MMRd (mismatch repair deficiency, equivalent to MSI-H), p53abn (abnormal p53, equivalent to CNH). NSMP (equivalent to copy-number low) is considered to have an intermediate prognosis overall, though it can be favorable in low-risk subgroups (e.g., ER-positive, low-grade tumors), especially in type I endometrial cancers [18,19]. While immunohistochemistry (IHC) for p53 and MMR proteins is relatively inexpensive, technically straightforward and widely available in most pathology departments worldwide, full molecular profiling including POLE exonuclease domain sequencing by next-generation sequencing (NGS) remains considerably more expensive and is currently not routinely available in many countries and healthcare settings, particularly in low- and middle-income regions. Therefore, several surrogate or simplified diagnostic algorithms have been proposed and are increasingly used in clinical practice when NGS is not feasible.

### 1.3. Endometrial Cancer Genetics

**Figure 3:**
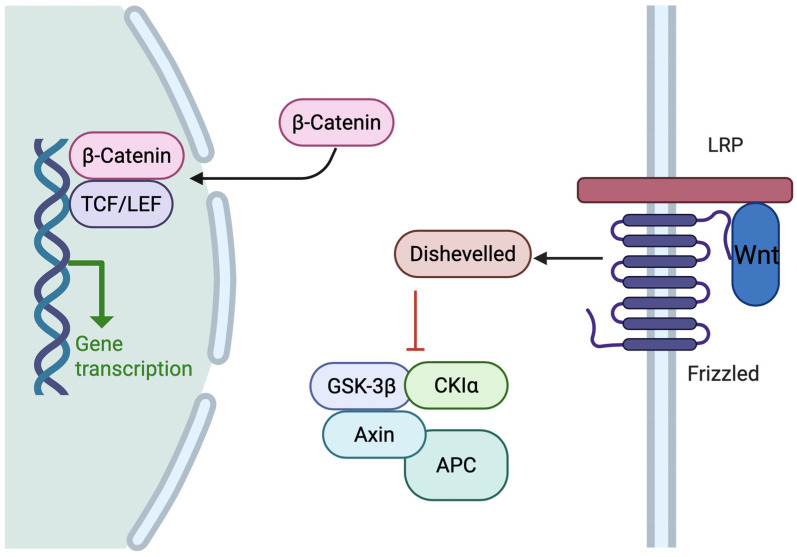
Schematic diagram of the Wnt/β-catenin signaling pathway. Binding of the Wnt ligand to Frizzled receptors and the LRP cofactor leads to activation of the Dishevelled protein, inhibition of the destructive complex (GSK-3β, CK1α, Axin, APC), stabilization of β-catenin, its translocation to the nucleus, and activation of target gene transcription (including through a complex with TCF/LEF). The curved arrow from Dishevelled pointing toward the destruction complex (Axin/APC/GSK-3β/CK1α) indicates inhibition of the complex. The arrow from cytoplasmic β-catenin to the nucleus (green arrow to gene transcription) represents activation. The direct arrow from β-catenin to TCF/LEF (in the nucleus) shows binding and co-activation of transcription. Created with BioRender.com.

The dominant pathogenetic pathways are PI3K/AKT/mTOR, Wnt/β-catenin (Figure 3), and DNA repair mechanisms [20,21].

POLEmut (7–10% of cases, mainly endometrioid histology) has a very high mutation load (>100 mut/Mb) with stable microsatellites (MSS), excellent prognosis, high T-cell infiltration (TILs), and P286R and V411L hotspots. POLEmut lacks coexistence with *TP53* mutations [22,23,24]. In 2025, de-escalation of adjuvant treatment was initiated in this subtype [25]. MMRd/MSI-H has hypermutations (10–100 mut/Mb) caused by loss of expression of *MLH1*, *MSH2*, *MSH6*, or *PMS2* (*MLH1* hypermethylation or germline mutations such as Lynch syndrome). It is characterized by high TMB, many neoantigens, and a good response to immunotherapy, resulting in an intermediate prognosis [26,27]. p53abn shows overexpression or absence of p53 in IHC, *TP53* mutations (nonsense, frameshift), co-occurring *PIK3CA*, *PPP2R1A*, and *FBXW7* mutations. Low TMB means poor prognosis. Chemoradiotherapy (PORTEC-3) and HER2- and HRD-targeted therapies are used in treatment [28,29]. The PI3K/AKT/mTOR pathway has *PTEN*, *PIK3CA*, and *AKT1* (E17K—highly predictive for capivasertib) mutations. Inhibitors of this pathway (capivasertib, olaparib, sapanisertib + metformin) achieve an ORR of 25–38% in appropriately selected subgroups [30,31,32,33,34,35]. Other common alterations such as *CTNNB1* give a poorer prognosis. In the case of *ARID1A*, we observe a 30–40% increase in PD-L1 and MSI expression and a potential response to immunotherapy and EZH2 inhibitors [36,37].

### 1.4. Immunotherapy and Targeted Therapy

**Figure 4:**
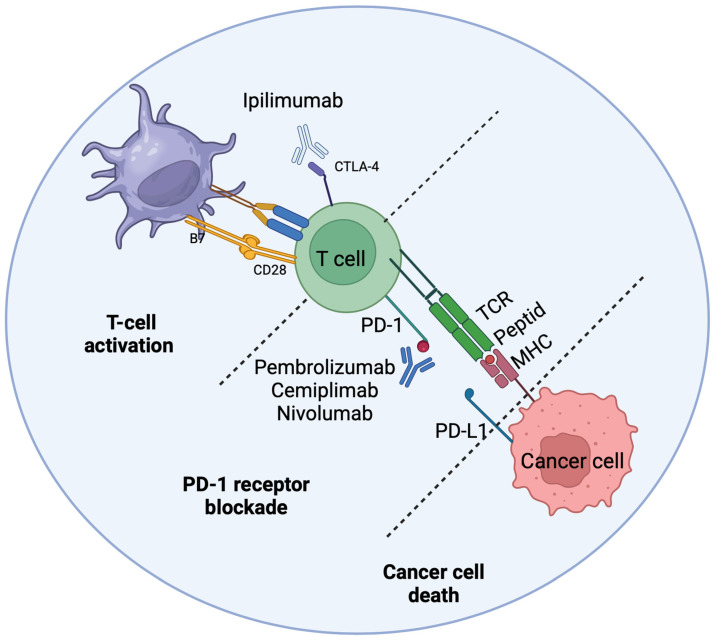
Mechanism of action of immune checkpoint inhibitors in endometrial cancer. Drugs such as pembrolizumab, nivolumab (anti-PD-1), durvalumab, atezolizumab, avelumab (anti-PD-L1), and ipilimumab (anti-CTLA-4) unblock T lymphocytes, enabling the recognition and destruction of cancer cells presenting antigens by MHC mechanism of action of immune checkpoint inhibitors in endometrial cancer. Dotted lines indicate the blocked/inhibited interactions caused by the respective monoclonal antibodies, leading to restored T-cell activity, proliferation, and ultimately cancer cell death. Created with BioRender.com.

Immune checkpoint inhibitors (ICIs), including pembrolizumab, dostarlimab, atezolizumab, avelumab, and retifanlimab, show high efficacy in tumors with mismatch repair deficiency (dMMR) or high microsatellite instability (MSI-H), achieving an objective response rate (ORR) of up to 60% (Figure 4) [38,39,40,41,42]. The dMMR/MSI-H status results from mutations or epigenetic silencing of mismatch repair (MMR) genes and leads to a high tumor mutational burden (TMB), which significantly increases tumor immunogenicity and sensitivity to ICIs. This mechanism is associated with the formation of neoantigens that are recognized by the immune system, facilitating the response to PD-1/PD-L1 axis blockade [40,41]. In most cases of EC, the efficacy of immunotherapy remains low. Hence, there is an urgent need to identify new predictive biomarkers. The combination of pembrolizumab or dostarlimab with chemotherapy significantly prolongs progression-free survival (PFS) in the dMMR/MSI-H subgroup. High tumor mutational burden (TMB ≥ 10 mut/Mb) further increases the predictive value of MMR status, highlighting the need for routine determination of both MMR and TMB to qualify patients for ICI treatment [43]. Germline mutations in MMR genes (e.g., in Lynch syndrome) and acquired *MLH1* promoter methylation differ in their response to immunotherapy, *MLH1*-methylated tumors typically show higher ORR, possibly due to a richer repertoire of neoantigens [44,45]. Neoadjuvant ICIs in dMMR endometrial cancer also show promising results in the preoperative period. For MMRp/MSS tumors, it is necessary to investigate new combinations, e.g., ICIs with PARP inhibitors, which may improve treatment efficacy [46,47,48,49,50]. Antibody-drug conjugates targeting HER2, especially trastuzumab deruxtecan, achieve ORR rates of 37.5–57.5% in patients with advanced EC and HER2 amplification/overexpression [51,52]. The mechanism of action of ADCs involves the selective delivery of a cytotoxic payload to HER2-overexpressing cells, which minimizes systemic toxicity while maximizing therapeutic effect [53]. For this reason, HER2 status testing by IHC and/or FISH should be standard in the selection of patients for this group of drugs, especially in advanced, high-risk forms of endometrial cancer, where classic chemotherapy often fails.

### 1.5. Hormone Therapy

Hormone therapies based on aromatase inhibitors (e.g., letrozole), CDK4/6 inhibitors (e.g., palbociclib), selective estrogen receptor modulators (e.g., imlunestrant), and selective estrogen receptor degraders (fulvestrant) are indicated in patients with ER-positive EC, which applies to most type I cases [54,55]. Hormone monotherapy in patients with ER-positive EC achieves an ORR of 20–30%, and the median progression-free survival (PFS) usually does not exceed 3–6 months. The combination of aromatase inhibitors with CDK4/6 inhibitors may extend the median PFS to 9–10 months in selected populations [54,55]. The mechanism of action of these drugs is to block the estrogen receptor pathway, which stimulates cancer cell proliferation in the presence of estrogen [56,57]. An additional benefit in this group of patients is the ability to monitor circulating tumor DNA (ctDNA). The ctDNA level correlates well with the response to hormonal treatment, enabling dynamic assessment of the effectiveness of therapy and early detection of minimal residual disease and subclinical progression. This makes it possible to quickly modify the therapeutic strategy even before the appearance of clinical symptoms or changes in imaging tests [58,59]. Progestogen therapies (targeted at the progesterone receptor, PR) show limited efficacy in endometrial cancer, indicating that PR expression alone is not a sufficiently strong predictive biomarker. The low efficacy of these drugs is also influenced by coexisting genetic and epigenetic aberrations. In young patients with early-stage endometrial cancer who wish to preserve their fertility, conservative treatment with a levonorgestrel-releasing intrauterine device (LNG-IUD) achieves a complete response rate of 70–82% [60,61]. In this specific clinical group, decisions regarding postoperative systemic treatment are currently based primarily on classic histopathological factors (tumor differentiation grade, depth of myometrial invasion, presence of lymph vascular invasion), and molecular and genomic biomarkers are not routinely used in the qualification process.

### 1.6. Prognostic Factors

*TP53* mutations and molecular subtypes of endometrial cancer are important prognostic factors and are crucial for the selection of a therapeutic strategy. The p53abn subtype, associated with *TP53* mutations and a high somatic copy number (CNH) profile, is characterized by an aggressive clinical course and the worst prognosis, with an overall survival (OS) of less than 50% [62,63]. In turn, the POLEmut subtype, which includes ultra-mutated tumors with mutations in the exonuclease domain of the POLE gene, is associated with an exceptionally favorable prognosis, with progression-free survival (PFS) exceeding 95% even in the metastatic stage. The MMRd/MSI-H subtype has an intermediate prognosis (worse than POLEmut, better than p53abn), while NSMP (copy-number low) is considered a subtype with a good prognosis, especially in type I endometrial cancers [64,65]. Molecular classification based on the TCGA study and its further clinical refinements (ProMisE, TransPORTEC) allow for the de-escalation of adjuvant treatment in the POLEmut subtype and the escalation of therapy in the p53abn subtype. *TP53* aberrations detected by IHC (overexpression, lack of expression, or abnormal cytoplasmic patterns) are an independent prognostic marker and indicate a benefit from intensified treatment (chemoradiotherapy) in high-risk cases [66]. The combination of molecular classification with the new FIGO 2023 system fundamentally changes the approach to endometrial cancer treatment, enabling truly personalized therapy and improved clinical outcomes.

### 1.7. Chemotherapy in the Age of Genetics

**Figure 5:**
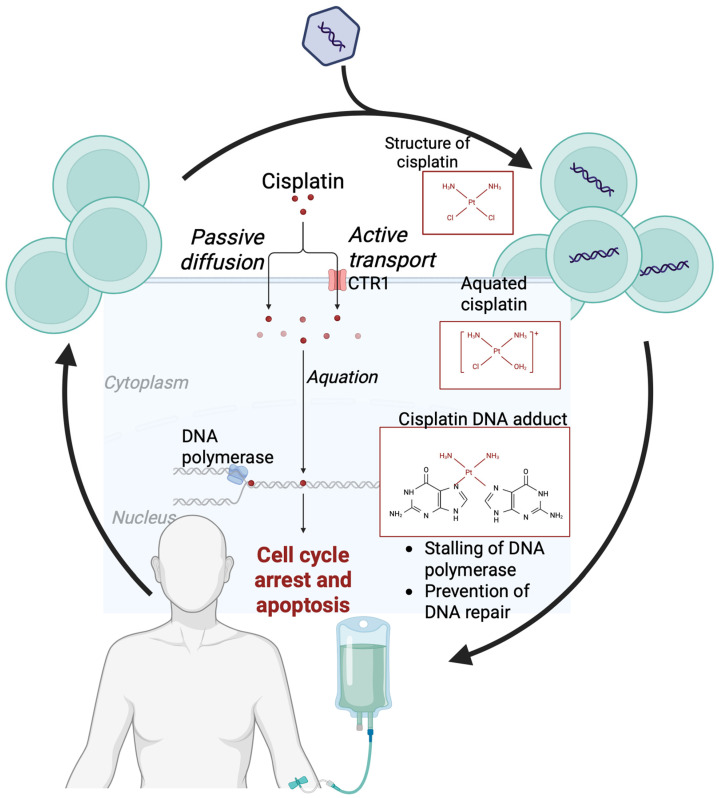
Mechanism of action of cisplatin. The drug enters the cell through passive diffusion and active transport with the participation of the CTR1 protein. In the cytoplasm, it undergoes aquation (replacement of chloride ions with hydroxyl groups), forms adducts with DNA, which leads to cell cycle arrest, inhibition of DNA replication and repair, and induction of apoptosis. Created with BioRender.com.

Chemotherapy (Figure 5) is often combined with other treatments, such as radiation therapy or immunotherapy. However, compared to immunotherapy, chemotherapy has very few validated genetic predictors of response. Mutations in the *TP53* gene are associated with a poorer prognosis in patients with endometrial cancer treated with chemotherapy, especially in combination with bevacizumab. Aberrant expression of the p53 protein in IHC is a recognized adverse prognostic factor [67]. Elevated Nrf2 expression, associated with oxidative stress pathways, correlates with a better response to concurrent chemoradiotherapy, but is not a sufficiently specific predictor to justify its use alone in clinical practice [68,69]. Most chemotherapy regimens used in endometrial cancer currently lack reliable genetic predictors of response, which significantly hinders the personalization of treatment [70]. There is an urgent need for further research into the molecular mechanisms of resistance and sensitivity to chemotherapy, which would allow the identification of predictive biomarkers and improve patient selection.

### 1.8. New Biomarkers and Translational Research

New biomarkers discovered in molecular and translational research have the potential to significantly improve treatment and prognosis in EC. Mutations or loss of *PTEN* function activate the PI3K/AKT/mTOR pathway and are associated with potential sensitivity to inhibitors of this pathway [71,72]. *ARID1A* mutations lead to increased PD-L1 expression and microsatellite instability (MSI), suggesting a possible benefit from checkpoint inhibitor immunotherapy and EZH2 inhibitors [73]. *DKK1* expression is being investigated as a potential predictive biomarker for response to the monoclonal antibody DKN-01 and may represent a new therapeutic option in patients with high expression of this protein. Long non-coding RNAs (lncRNAs) are associated with endometrial cancer progression and may be predictors of response to immunotherapy, which requires further research in this area [74]. MicroRNAs (miRNAs) represent another promising class of biomarkers in EC, with potential applications in early diagnosis, prognosis, and prediction of treatment response. Dysregulated expression of miRNAs, particularly the miR-200 family, miR-205, and miR-21, has been consistently reported in EC tissues and circulating in plasma/serum, enabling non-invasive detection [75,76,77,78]. Notably, several miRNAs altered in endometriosis overlap with those in endometrioid EC, suggesting their utility in identifying patients at risk of malignant transformation [79]. Ongoing translational studies explore miRNA panels as companions for immunotherapy and targeted therapies, warranting further validation in prospective cohorts.

The aim of this paper is to review current clinical and preclinical studies on the impact of genetic and molecular changes in endometrial cancer on the effectiveness of treatments. Emphasis was placed on assessing the extent to which genetic and protein markers currently allow for the personalization of treatment, and on identifying directions for future research on new therapies and biomarkers.

## 2. A Review of the Literature

PubMed and Web of Science databases were searched for publications on the role of genetic and molecular changes in the diagnosis, treatment, and prognosis of endometrial cancer. The main part of the review consists of phase I–III clinical trials published between 2020 and 2025.

### 2.1. Immunotherapy

Immunotherapy (Table 1) is playing an increasingly important role in the treatment of endometrial cancer, especially in tumors with mismatch repair deficiency (dMMR) and high microsatellite instability (MSI-H). Checkpoint inhibitors (mainly pembrolizumab and dostarlimab) show high efficacy in this group of patients, significantly prolonging progression-free survival and overall survival compared to conventional chemotherapy.

### 2.2. Targeted Therapy

Results of targeted therapy (Table 2).

### 2.3. Hormone Therapy Studies

Endometrial cancer hormone therapy (Table 3) is mainly used in hormone-dependent tumors, especially those expressing estrogen and progesterone receptors.

### 2.4. Chemotherapy

Chemotherapy (Table 4) remains a cornerstone of treatment for EC, particularly in adjuvant settings for high-risk disease or advanced/recurrent stages (Figure 5). It is often combined with radiotherapy, immunotherapy, or targeted agents to enhance efficacy. However, unlike immunotherapy or targeted therapies, chemotherapy has fewer validated genetic predictors, limiting personalization. This section reviews key molecular factors influencing response and highlights the need for further biomarker research. Mutations in the TP53 gene are linked to poorer prognosis in EC patients receiving chemotherapy, especially when combined with bevacizumab, with aberrant p53 expression via immunohistochemistry serving as an adverse prognostic factor [67]. For instance, in a phase 2 trial, TP53 mutations or p53 overexpression correlated with worse progression-free survival without bevacizumab, but predicted greater benefit when added [116]. Elevated Nrf2 expression, tied to oxidative stress pathways, is associated with better responses to concurrent chemoradiotherapy but lacks specificity for routine use [68,69].

### 2.5. Molecular Classification and Prognosis

The molecular classification of endometrial cancer according to The Cancer Genome Atlas (TCGA) and its practical implementation ProMisE (POLE-ultra mutated, MMRd, p53-abnormal, NSMP/no specific molecular profile) is of key prognostic and predictive importance. Horeweg et al. [120], Clements et al. [121], and the results of the PORTEC-3 [122] clearly showed that the p53abn subtype has the worst prognosis (HR 2.14 for recurrence in PORTEC-3). The POLE-ultra mutated subtype is associated with the best prognosis, even in cancers with a high degree of histological malignancy. Fremond et al. [123] developed a deep learning model that accurately predicts molecular subtype based on histopathological and immunohistochemical data. Bogani et al. [124] demonstrated that co-occurring *TP53* and *PTEN* mutations are an independent risk factor for lymph node involvement.

### 2.6. New Therapeutic Approaches

Cassier et al. [125] demonstrated in preclinical models that netrin-1 blockade (using the NP137 antibody) inhibits tumor growth and epithelial–mesenchymal transition (EMT) in endometrial cancer with *PTEN* loss. Piffoux et al. [126] evaluated the triplet of PARP inhibitor (olaparib) with metronomic cyclophosphamide and metformin in recurrent advanced endometrial cancer (ENDOLA phase I/II trial). Efficacy was limited (ORR 21.4%), with no reliable predictive biomarkers identified.

### 2.7. Other Biomarkers and Factors

Deng et al. [127] suggest that regular physical activity may beneficially modulate immune system function in carriers of Lynch syndrome-associated mutations, although direct data on endometrial cancer are lacking. Bendifallah et al. [128] identified a salivary microRNA signature (109 miRNAs) for the diagnosis of endometriosis (AUC 0.95), but its relevance to endometrial cancer remains unexplored.

## 3. Discussion

EC is one of the most common gynecological cancers, and its molecular heterogeneity presents both an obstacle and an opportunity for the use of precise therapeutic tactics. Advances in genetics and molecular biology have led to the identification of key biomarkers such as dMMR, MSI-H, *TP53* mutations, POLE, HER2 amplification, alterations in the PI3K/AKT/mTOR pathway, and *ARID1A* mutations, as well as their significance in determining treatment response and prognosis. In recent years, EC treatment has entered a whole new era with the introduction of immune checkpoint inhibitor (ICI) immunotherapy, specifically pembrolizumab, durvalumab, atezolizumab, avelumab, and retifanlimab. Studies have shown that the efficacy of ICIs is closely related to dMMR/MSI-H status due to the high immunogenicity of these tumors. The efficacy of pembrolizumab in combination with chemotherapy in stage II-IV dMMR tumors was found to be very significant according to Eskander et al. [80], PFS was significantly prolonged (HR 0.30) compared to the pMMR group (HR 0.64), making MMR status a key covariate. Similar results were obtained for dostarlimab in combination with chemotherapy, with a clear effect on PFS in the dMMR/MSI-H population (HR 0.28) and no benefit in pMMR/MSS. O’Malley et al. [82] reported an ORR of 48% in MSI-H EC treated with pembrolizumab. All of this points to MMR loss, both due to mutation and epigenetic silencing. Consistent with earlier observations, Marabelle et al. [89] confirmed that high TMB (10+ mutations/Mb) correlates with a 29% ORR for pembrolizumab and with the MSI-H phenotype. Other factors influencing ICI efficacy include Lynch syndrome associated with germline MMR gene mutations and Lynch-like tumors associated with spontaneous dMMR leading to *MLH1* methylation. Bellone et al. [48] and Ettorre et al. [97] also reported increased ORR levels in Lynch-like tumors compared to *MLH1* methylation cases treated with pembrolizumab, which may be related to differences in neoantigen levels or the dynamics of the immune microenvironment. Deng et al. [127] observed that exercise strengthens the immune system in patients with Lynch syndrome, and therefore synergy between lifestyle and immunotherapy is possible. However, there are no specific data for EC. In other ICIs (atezolizumab [80], avelumab: Pignata et al. [86] and retifanlimab: Berton et al. [94]), it is also administered as high, dMMR/MSI-H (ORR 42–48%). The study by Rubinstein et al. [129] did not identify any specific predictors for durvalumab with tremelimumab, which further highlights the difficulty of approaching MMRp/MSS EC, given that it accounts for most cases. Since neoadjuvant ICIs, as found by Eerkens et al. [92], had an ORR of 60% in dMMR EC, immunotherapy may play a promising role in preoperative management. The efficacy of combinations such as dostarlimab and niraparib [98] has an ORR of 33% in dMMR but will require more genetic profiling to increase efficacy. New immunotherapies such as anti-GITR antibodies [130] and the anti-TIM-3 combination [99] provide little EC-specific data and require further molecular characterization. Knisely et al. [131] and Patel et al. [132] investigated the predictive role of avelumab in combination with other immunomodulatory biomarkers, but there are no specific genetic markers that would enable this. Drugs are specifically targeted to the type of molecular abnormality, e.g., HER2 amplification, PI3K/AKT/mTOR pathway mutation, *ARID1A* mutation, or *KRAS* mutation, and provide narrowly specific intervention in target EC subtypes. HER2 amplification or overexpression is particularly relevant in serous and sarcomatous subtypes. In HER2-amplified EC, the ORR after treatment with trastuzumab and deruxtecan was 45% according to Yagisawa et al. [105] and 57.5% according to Oaknin et al. [106] in HER2 IHC 3+ cases. The demonstration by Lumish et al. [108] in EC with HER2 overexpression confirmed the importance of HER2 amplification or high IHC expression as a predictive biomarker and showed an ORR of 37.5%. An example of the importance of routine HER2 testing in EC and aggressive EC is the efficacy of trastuzumab-based antibody conjugates such as deruxtecan. Another important target is the PI3K/AKT/mTOR pathway, which can often be modified by EC (mutations in *PTEN*, *PIK3CA*, and *AKT1*). In a study by Kalinsky et al. [103], 2/5 patients with EC with an *AKT1* mutation at position 17, replacing glutamic acid with lysine, responded partially to treatment with capivasertib, demonstrating the specificity of the mutation’s efficacy. In EC, the use of the PI3K/AKT pathway appears to be associated with an ORR of olaparib/capivasertib of 25% [104], and therefore activity is reported in molecularly defined cohorts. Subbiah et al. [110] verified mTOR/AKT/PI3K modifications that are predictors of sapanisertib + metformin efficacy in EC patients, indicating that pathway inhibitors will be valuable EC drugs in the future. Studying sotorasib in tumors with *KRAS* G12C mutation, Hong et al. [102] provided several insights into EC that suggest that *KRAS* mutations are not as common in EC as in other diseases. Keller et al. [107] demonstrated that *ARID1A* mutations predispose EC to inhibition of EZH2 by tulmimetostat, thereby expanding the clinical prospects of endometrioid EC, in which *ARID1A* mutations are common. Makker et al. [100] tested lenvatinib with pembrolizumab, recording an ORR of 31.9–38. MMR status played an important role in modifying response in this study, although specific mutation combinations were not the best predictor of response. As Arend et al. [74], *DKK1* expression correlated with response to DKN-01 (*DKK1*-high ORR 25%) and is therefore considered an innovative biomarker. In the case of serous uterine cancer, Liu et al. [133] tested ada-vosertib, but the lack of specific biomarkers prevented them from drawing conclusions. Konstantinopoulos et al. [111] reported an ORR of 30% in recurrent ER-positive EC using letrozole and abemaciclib. The study by Mirza et al. [55] showed that PFS in ER-positive EC patients using palbociclib and letrozole was 8.3 months, demonstrating the importance of ER status. ORR for imlunestrant with or without abemaciclib was 22% and 20%, respectively, according to Jhaveri et al. [113] and Yonemori et al. [114], with both ER status and RB1 presented as predictive factors. Further evidence for the activity of this combination is the 44% ORR achieved by Green et al. [115] using fulvestrant plus abemaciclib in hormone receptor-positive advanced or recurrent endometrial cancer. In PR-positive EC, Andres et al. [112] did not observe much activity in response to ER onapristone, even though PR expression is required in EC, highlighting that PR alone may not be a good predictor and that other genetic or epigenetic factors influencing treatment response should be sought. In early-stage EC, Westin et al. [104] used a levonorgestrel intrauterine device with an 82% response rate. However, as there are no genetic biomarkers, hormonal treatment at this site is limited to histopathological characteristics. Research on chemotherapy, which usually involves various modalities, uses fewer genetics-related mechanisms compared to immunotherapy or targeted therapies. This was demonstrated by Thiel et al. [116], who demonstrated an association between *TP53* mutation in the context of poorer PFS (HR 1.8) after bevacizumab and chemotherapy, with p53 immunohistochemistry (IHC) as a prognostic factor. Bae-Jump et al. [117] and Kristeleit et al. [118] showed that it was not possible to distinguish between the individual genetic biomarkers of paclitaxel/carboplatin with metformin and doxorubicin with lurbinectedin, suggesting that treating cancer as a disease with specific genetic conditions is not always promising. Leary et al. [119], in whom ibrilatazer and paclitaxel/carboplatin had an ORR of 35%, reported no changes in the PI3K/AKT/mTOR pathway as a predictor. According to Matulonis et al., there are no specific biomarkers for response to cisplatin-based chemotherapy with radiotherapy or to carboplatin and paclitaxel [134], so it appears that additional research on molecular predictors of response to chemotherapy is needed. Other methods studied, such as molecular and translational studies, have proven beneficial in studying EC classification and its impact on prognosis. In PORTEC, Vermij et al. [122] and Fremond et al. [123] confirmed that prognosis can be predicted based on molecular subtype (POLEmut, MMRd, p53abn), with P53ABN predicting the worst outcome. These results were confirmed by Horeweg et al. [120] in early-stage EC, who noted that molecular profiling plays a key role. As demonstrated by Bogani et al. [124], molecular features such as *TP53* and *PTEN* are associated with the possibility of lymph node involvement and are considered in treatment planning. Cassier et al. [125] investigated the consequences of Netrin-1 blockade in preclinical models and indicated that the growth of *PTEN*-deficient EC cells was inhibited and is not dispensable, hence a new therapeutic option. A study by Li et al. [135] showed that the presence of lncRNAs involved in disulfide glycosylation has prognostic value, and the response to immunotherapy opens opportunities for further research. According to Piffoux et al. [126] and Ahmed et al. [136], no specific biomarkers associated with olaparib/cyclophosphamide/metformin or temsirolimus/metformin combinations have been identified and require further investigation. Bendifallah et al. [128] investigated the microRNA signature in endometriosis, but conclusions are limited due to the lack of direct EC data. Such a finding has important clinical implications. TMB and MMR are important predictors of ICI response and thus become a benchmark for immunotherapy eligibility. The study of alterations in Her2, ER, Rb1, and PI3K/AKT pathways is important for targeted and endocrine therapy, particularly in serous and endometrioid subtypes. The prognostic value of *TP53* mutations and molecular classification (*POLE*, MMRd, p53abn) can be used for therapeutic decisions, especially in adjuvant scenarios. There is still a challenge in treating MMRp/MSS tumors, which have a low ICI response. Newer biomarkers, namely *DKK1*, lncRNA, or epigenetics-based biomarkers, may help improve outcomes in this patient group. ctDNA monitoring may be a useful decision-making tool, especially during hormone therapy. Combining multi-omics data will provide a complete molecular picture of EC, and combination therapies, such as ICI with PARP inhibitors or any targeted therapy, may optimize the activity of the heterogeneous EC entity. Genetic factors of EC, such as MMR status, TMB, *TP53*, mutations in *POLE*, HER2, *PTEN*, PI3K/AKT, and *ARID1A* genes, are essential for treatment response and outcome. Immunotherapy can be highly effective in dMMR/MSI-H malignancies, while targeted therapy can potentially work in malignancies with HER2 amplification and PI3K/AKT mutation, and hormone therapy is effective in estrogen receptor (ER)-positive EC. Current advances include molecular profiling as an essential component of personalized EC treatment, and research into new biomarkers and combinations of new therapies to achieve better clinical outcomes, particularly in problematic MMRp/MSS subtypes, is also important. Further research focusing on the immune microenvironment and its role in interactions with genetic alterations may reveal new potential treatment options that could change the management of EC in the next few years.

## 4. Future Perspective

In the long term, the integration of artificial intelligence (AI) and new technologies has potential in the treatment of EC, enabling more precise diagnosis, prognosis, and personalization of therapy. AI-based predictive models using machine learning algorithms on histopathological preparations, radiomic features from MRI/CT imaging, and multi-omic data (genomics, transcriptomics, proteomics), have the potential to surpass traditional methods in classifying subtypes, assessing recurrence risk, and predicting treatment response, especially in the case of difficult-to-treat pMMR/MSS cancers [137,138,139,140]. For example, convolutional neural networks (CNN) using deep learning have shown high accuracy (AUC > 0.90) in distinguishing EC molecular subtypes based on routine H&E-stained preparations, potentially improving ESGO/ESTRO/ESP risk stratification and aiding real-time adjuvant therapy decisions in clinical settings. New technologies such as single-cell RNA sequencing (scRNA-seq) and spatial transcriptomics combined with artificial intelligence, will unravel the heterogeneity of the tumor immune microenvironment, identifying new neoantigen profiles or immune evasion signatures that could increase the efficacy of ICIs in non-immunogenic EC subtypes [141,142,143,144]. These advances, if confirmed in prospective studies, could transform the care of EC patients toward fully AI-assisted precision oncology, reduce overtreatment and improve survival in high-risk populations.

## 5. Limitations

Molecular heterogeneity of EC, and particularly intratumor heterogeneity, is a major problem for individual treatment. Subtypes that are rare require larger study groups to better understand how subtypes work and respond to treatment. The lack of predictive response factors among MMRp/MSS tumors and the low number of biomarkers of chemotherapy activity indicate the need to investigate the mechanism of resistance and novel drug targets. NGS and high prices of advanced molecular assays, as well as lack of accessibility in some areas, continue to be obstacles to the final genetic profiling of masses. There are also ethnic differences in the projections, such as increased mortality rates for African American women, indicating the need for a more inclusive clinical trial. Deep learning models and other types of artificial intelligence (AI) have shown encouraging results in predicting molecular subtypes in histology based on morphological images (H&E), which could make diagnosis more affordable and more widely available. In future studies, efforts should be made to incorporate multi-omics datasets (genomics, transcriptomics, proteomics) to develop detailed molecular profiles of ECs and to incorporate combination therapy, e.g., with ICI and PARP inhibitors, PI3K/AKT inhibitors or ADCs. These strategies would be able to optimize overall treatment outcomes in heterogeneous EC subtypes and in patients with poor prognosis.

## 6. Conclusions

Genetic factors in endometrial cancer, such as MMR, high TMB, *TP53*, *POLE*, HER2 amplification, PI3K/AKT alteration, *ARID1A* mutation, and new biomarkers *DKK1* and lncRNA, are crucial for treatment planning and prognosis. Immunotherapy has shown very good results in dMMR/MSI-H tumors, with a very good ORR rate, better PFS, and is the first-line treatment in this population. The efficacy of targeted drugs, especially those with HER2 and PI3K/AKT pathway, is promising in molecularly defined categories, namely serous and endometrioid EC. ER-positive responds to hormonal treatment, and ER and RB1 predictors and ctDNA are biomarkers. Chemotherapy is widely used, but there are few genetic predictors, and *TP53* mutations are associated with a poorer prognosis. Classification by EC molecular type results in a change in EC treatment (POLEmut, MMRd, p53abn, NSMP), which will ensure personalized treatment and even better outcomes. Challenges such as the treatment of MMRp/MSS cancers and molecular heterogeneity, as well as access to genetic testing, must be addressed. The future includes the development of new biomarkers and the investigation of combination therapies and synergies to increase efficacy in all types of EC. Further research into the immune microenvironment and its relationship to genetic alterations may provide new therapeutic targets, and the coming years may bring changes in the treatment of EC.

## Figures and Tables

**Table 1:** Application of immunotherapy.

Authors	Year	Type of Study	Drug/Therapy	Diagnosis	Request
Eskander RN et al. [80]	2023	Phase 3, randomized	Pembrolizumab + Chemotherapy	Advanced EC	Better PFS in dMMR (HR 0.30) and pMMR (HR 0.54); MMR status crucial to the response.
Mirza MR et al. [81]	2023	Phase 3, randomized	Dostarlimab + Chemotherapy	Primary advanced or recurrent EC	Significant improvement in PFS in dMMR/MSI-H (HR 0.28, 95% CI 0.16–0.50) and in overall population (HR 0.64); modest but statistically significant PFS benefit also in MMRp/MSS subgroup (HR 0.76, 95% CI 0.60–0.96); strongest predictive value of MMR/MSI status.
O’Malley DM et al. [82]	2022	Phase 2, single arm	Pembrolizumab	Advanced MSI-H EC	ORR 48% in MSI-H; MMR deficiency highly predictive of response.
Oaknin A et al. [83]	2022	Phase 1, single arm	Dostarlimab	dMMR/MSI-H or MMRp/MSS EC	ORR 42.3% in dMMR/MSI-H vs. 13.4% in MMRp/MSS; MMR status is crucial.
Eskander RN et al. [84]	2025	Phase 3, randomized	Pembrolizumab + Chemotherapy	Advanced or recurrent EC	PFS Benefit in dMMR (HR 0.30), OS trend favorable; MMR status is crucial.
Colombo N et al. [85]	2024	Phase 3, randomized	Atezolizumab + Chemotherapy	Advanced or recurrent EC	PFS benefit in dMMR (HR 0.36); Predictive MMR status.
Pignata S et al. [86]	2024	Phase 3, randomized	Avelumab	First-line EC	Phase 3 trial conducted exclusively in dMMR patients; PFS benefit with avelumab + carboplatin-paclitaxel vs. chemotherapy alone in dMMR first-line endometrial cancer (HR 0.42, 95% CI 0.25–0.70).
Maio M et al. [87]	2022	Phase 2	Pembrolizumab	MSI-H/dMMR Tumors	ORR 49.4% in MSI-H endometrial cancer; MMR deficiency is crucial.
Powell MA et al. [88]	2025	Phase 3, randomized	Dostarlimab + Chemotherapy	dMMR/MSI-H advanced or recurrent EC	Phase 3 (RUBY part 2) in dMMR/MSI-H primary advanced/recurrent endometrial cancer; median PFS not reached with dostarlimab + chemotherapy vs. 14.7 months with placebo + chemotherapy (HR 0.28, 95% CI 0.16–0.50); highly predictive MMR/MSI status.
Marabelle A et al. [89]	2020	Phase 2	Pembrolizumab	Advanced solid tumors	High TMB (≥10 mut/Mb) associated with an ORR of 29%; TMB predictive in MSI-H cases.
Slomovitz BM et al. [90]	2025	Phase 3, randomized	Pembrolizumab + Adjuvant chemotherapy	High-risk EC	PFS benefit in dMMR (HR 0.31); MMR status is crucial.
André T et al. [91]	2023	Non-randomized, controlled	Dostarlimab	Solid dMMR tumors	ORR 38.7% in dMMR endometrial cancer; Predictive MMR status.
Eerkens AL et al. [92]	2024	Phase 1	Neo-adjuvant checkpoint blockade	dMMR EC	ORR 60% in dMMR; Predictive MMR status.
O’Malley DM et al. [93]	2025	Phase 2	Pembrolizumab	MSI-H/dMMR and non-MSI-H EC	ORR 46% in MSI-H vs. 14% in non-MSI-H; MMR status is crucial.
Berton D et al. [94]	2024	Phase 1	Retifanlimab	dMMR/MSI-H EC	ORR 48%; Predictive MMR status.
Bellone S et al. [48]	2021	Phase 2	Pembrolizumab	Recurrent MSI-H EC	ORR higher in Lynch-like vs. *MLH1*-methylated; MMR and Lynch-like predictive status.
Oaknin A et al. [95]	2020	Phase 1, non-randomized	Dostarlimab	Recurrent or advanced dMMR EC	ORR 42%; Predictive MMR status.
Antill Y et al. [96]	2021	Phase 2, non-randomized	Durvalumab	Advanced EC dMMR and MMRp	ORR higher in dMMR; Predictive MMR status.
Ettorre VM et al. [97]	2025	Phase 2	Pembrolizumab	Recurrent MSI-H EC	ORR higher in Lynch-like; MMR and Lynch-like predictive status.
Madariaga A et al. [98]	2023	Phase 2	Niraparib ± Dostarlimab	Recurrent EC	ORR 33% in dMMR; Predictive MMR status.
Hollebecque A et al. [99]	2021	Phase 1	α-PD-L1 ± α-TIM-3	MSI-H/dMMR neoplasms	ORR in MSI-H; Predictive MMR status.

**Table 2:** Results of targeted therapy.

Authors	Year	Type of Study	Drug/Therapy	Diagnosis	Request
Makker V et al. [100]	2023	Phase 3, randomized	Lenvatinib + Pembrolizumab	Previously treated advanced EC	ORR 32.4% in pMMR/MSS and 50.6% in MSI-H/dMMR; MMR status predicts greater benefit in dMMR subgroup.
Makker V et al. [101]	2020	Phase 1b/2	Lenvatinib + Pembrolizumab	Advanced EC	ORR 38%; no specific genetic biomarkers, but MMR status noted.
Hong DS et al. [102]	2020	Phase 2	Sotorasib	Advanced solid tumors	Limited data for endometrial cancer; *KRAS* G12C mutation required for inclusion.
Kalinsky K et al. [103]	2021	Non-randomized	Capivasertib	Cancers with *AKT1* E17K mutation	PR in 2/5 of patients with endometrial cancer with *AKT1* E17K; mutation-specific response.
Westin SN et al. [104]	2021	Phase 1b	Olaparib + Capivasertib	Recurrent EC	ORR 25%; response-related changes in the PI3K/AKT pathway.
Yagisawa M et al. [105]	2024	Phase 2, Basket Study	Trastuzumab Deruxtecan	HER2 amplified solid tumors	ORR 45% in HER2-amplified endometrial cancer; HER2 predictive amplification.
Oaknin A et al. [106]	2024	Post Hoc Analysis	Trastuzumab Deruxtecan	HER2-Expressing Solid Tumors	ORR 84.6% in endometrial cancer with HER2 IHC 3+ (57.5% overall in HER2-expressing endometrial cohort); HER2 expression is crucial.
Keller PJ et al. [107]	2024	Preclinical/translational study	Tulmimetostat	Cancers with *ARID1A* mutation	*ARID1A* mutations sensitized endometrial cancer to EZH2 inhibition.
Lumish M et al. [108]	2024	Phase 2	Zanidatamab	EC with HER2 overexpression	Clinical benefit rate (CBR) 37.5% (SD ≥ 24 weeks); ORR 6.2% (1 PR); HER2 overexpression.
Backes FJ et al. [109]	2021	Phase 1	Lenvatinib + Paclitaxel	Recurrent EC	ORR 50%; lack of specific genetic biomarkers.
Subbiah V et al. [110]	2024	Phase 1	Sapanisertib + Metformin	Advanced solid tumors	Response-related changes in the mTOR/AKT/PI3K pathway.
Arend R et al. [74]	2023	Phase 2, Basket Study	DKN-01	Recurrent EC	*DKK1* predictive for response; ORR 25% in *DKK1*-high

**Table 3:** Summary of the use of hormone therapy.

Authors	Year	Type of Study	Drug/Therapy	Diagnosis	Request
Konstantinopoulos PA et al. [111]	2023	Phase 2, two-stage	Letrozole + Abemaciclib	Recurrent ER-positive EC	ORR 30%; ER expression and RB1 status predictive for responses.
Mirza MR et al. [55]	2025	Phase 2, randomized	Palbociclib + Letrozole	Advanced/recurrent ER-positive EC	PFS 8.3 months in ER-positive; ER status crucial.
Andres S et al. [112]	2024	Basket Survey	Onapristone ER	PR-positive EC	Limited response; PR expression required, absence of specific mutations.
Jhaveri KL et al. [113]	2024	Phase 1a/1b	Imlunestrant ± Abemaciclib	ER-positive BC	Phase 1a/1b EMBER study in ER+/HER2- advanced breast cancer (no endometrial cohort); ORR 22% in breast cancer monotherapy arm; predictive ER status in breast cancer.
Yonemori K et al. [114]	2024	Phase 1a/1b	Imlunestrant ± Abemaciclib	ER-positive EC	ORR 20%; ER expression and RB1 status are predictive.
Green AK et al. [115]	2025	Phase 2	Fulvestrant + Abemaciclib	HR-positive EC	ORR 44%; responses predominantly in copy number-low/no specific molecular profile tumors.

**Table 4:** Results of chemotherapy.

Authors	Year	Type of Study	Drug/Therapy	Diagnosis	Request
Thiel KW et al. [116]	2022	Phase 2	Bevacizumab + Chemotherapy	EC	*TP53* mutations/p53 IHC overexpression associated with worse PFS overall (HR ~1.8 without bevacizumab); p53 status predictive for greater benefit with bevacizumab addition (PFS HR 0.41, OS HR 0.28 in TP53 mut/p53 OE subgroup vs. temsirolimus arm).
Bae-Jump VL et al. [117]	2025	Phase 2/3	Paclitaxel/Carboplatin + Metformin	Stage III/IV or recurrent EC	Lack of genetic biomarkers; no significant benefit of metformin.
Kristeleit R et al. [118]	2021	Phase 1	Doxorubicin + Lurbinectedin	Advanced EC	ORR 44%; lack of specific genetic biomarkers.
Leary A et al. [119]	2024	Phase 1/2	Ibrilatazar + Paclitaxel/Carboplatin	Advanced/recurrent EC	ORR 65.8% (13.2% CR, 52.6% PR); AKT/mTOR pathway inhibition crucial for mechanism (induces cytotoxic autophagy); PD biomarkers confirmed pathway engagement.

## Data Availability

All data has been included.

## Abbreviations

The following abbreviations are used in this manuscript:

ADC	Antibody-drug conjugate
AKT1	AKT serine/threonine kinase 1
ARID1A	AT-rich interaction domain 1A
CDK4/6	Cyclin-dependent kinase 4/6
CNH	Copy-number high
CNL	Copy-number low
ctDNA	Circulating tumor DNA
CTNNB1	Catenin beta 1 (gen)
dMMR	Deficient mismatch repair
DKK1	Dickkopf WNT signaling pathway inhibitor 1
EC	Endometrial cancer
ERFBXW7	Estrogen receptorF-box and WD repeat domain containing 7
FIGO	International Federation of Gynecology and Obstetrics
FISH	Fluorescence in situ hybridization
GSK-3β	Glycogen synthase kinase 3 beta
HER2	Human epidermal growth factor receptor 2
HRD	Homologous recombination deficiency
ICI	Immune checkpoint inhibitor
IHC	Immunohistochemistry
KRAS	Kirsten rat sarcoma viral oncogene homolog
lncRNA	Long non-coding RNA
LNG-IUD	Levonorgestrel-releasing intrauterine device
LRP	LDL receptor related protein
MLH1	MutL homolog 1
MMR	Mismatch repair
MMRd	Mismatch repair deficient
MMRp	Mismatch repair proficient
MSH2	MutS homolog 2
MSH6	MutS homolog 6
MSI	Microsatellite instability
MSI-H	Microsatellite instability-high
MSS	Microsatellite stable
mTOR	Mammalian target of rapamycin
NGS	Next-generation sequencing
NSMPORR	No specific molecular profileObjective response rate
OS	Overall survival
PCOS	Polycystic ovary syndrome
PD-1	Programmed cell death protein 1
PD-L1	Programmed death-ligand 1
PFS	Progression-free survival
PI3K	Phosphoinositide 3-kinase
PIK3CA	Phosphatidylinositol-4,5-bisphosphate 3-kinase catalytic subunit alpha
PMS2	PMS1 homolog 2
POLE	DNA polymerase epsilon
POLEmut	POLE mutated
PPP2R1A	Protein phosphatase 2 scaffold subunit A alpha
PR	Progesterone receptor
PTEN	Phosphatase and tensin homolog
p53abn	Abnormal p53
pMMR	Proficient mismatch repair
TCGA	The Cancer Genome Atlas
TILs	Tumor-infiltrating lymphocytes
TMB	Tumor mutational burden
TP53	Tumor protein p53
